# Enhancement on thermal, electrical and waterproof performances of cellulose insulating paper with Ar/OMCTS/BN plasma

**DOI:** 10.1038/s41598-026-57750-5

**Published:** 2026-06-10

**Authors:** Lei Zhu, Honghua Xu, Rui Chen, Can Zhang, He Wang, Yong Li, Li Xu, Biao Wang, Ziqiang Xu

**Affiliations:** State Grid Nanjing Power Supply Company, Nanjing, China

**Keywords:** Cellulose insulating paper, Plasma polymerization, Thermal conductivity, Dielectric adaption, Waterproof performance, Energy science and technology, Engineering, Materials science

## Abstract

Cellulose paper plays the key role in the oil-paper insulation system ensuring safe and reliable operation of power systems, which faces challenges related to its inherent low thermal conductivity, high dielectric constant and hydrophilicity. Herein, polymerization reactions are induced on the surface and within the bulk of the cellulose insulating paper via atmospheric-pressure low-temperature plasma with a precursor formed by incorporating BN powders into octamethylcyclotetrasiloxane (OMCTS). As indicated by SEM, EDS, FTIR, and XPS results, a low-polarity cross-linking network is constructed within intrinsic porous structures of the paper, comprehensively improving thermal, electrical and waterproof performances. The thermal conductivity is increased by 15.9%, thereby enabling a significant enhancement in the heat dissipation capability. The relative dielectric constant of the insulating paper is decreased by 27.3%, maintaining effective dielectric adaptation with insulating oil. The surface and bulk insulation performance of the oil-impregnated paper is reinforced, with a 9.4% increase in the flashover voltage and a 17.5% improvement in bulk breakdown strength. The wetting property is changed from hydrophilic to hydrophobic, reducing water absorption by 32.6%, inhibiting the transport of water molecules. This work provides a promising approach for developing high-performance insulating paper for safe and stable power transmission.

## Introduction

The oil-paper insulation system is widely employed in high voltage power transmission, while its performance is constrained by several inherent limitations^1–3^. The relative dielectric constant of the insulating paper is typically double that of the oil, which distorts the electric field distribution and promotes charge transport, leading to insulation failure^4,5^. Furthermore, the thermal conductivity of the paper is reported to be below 0.7 W/(m·K), which impedes efficient heat dissipation, accelerates thermal aging, and even triggers electrical breakdown^6,7^. Additionally, due to the hydrophilic property of cellulose, water molecules in the system tend to migrate from the oil into the paper, degrading paper performance through hydrolysis^8^. Therefore, it is crucial to simultaneously reduce the relative dielectric constant, improve thermal conductivity and insulating strength, and suppress water absorption.

Enhancing the insulating paper performance is the key to improve the stability of oil-paper systems, since insulating paper cannot be recycled^9–12^. Researchers have employed several methods, such as nanoparticle doping, chemical grafting and surface treatment, to modify the insulating paper. Li et al. doped cellulose insulating paper with octaaminophenyl-polysilsesquioxane (OAPS) nanoparticles, which reduced the relative dielectric constant by 16.5%^13^. Mo et al. grafted OAPS onto paper, reducing the dielectric constant by 18.8% and decreasing dielectric loss by 37.5%^14^. Wu et al. deposited SiO_2_ film on ceramic fiber paper, enhancing the DC surface flashover voltage by 15.1%^15^. Although some insulating paper properties could be improved by the above methods, it remains a challenge to decrease dielectric constant, improve insulation and mechanical strength simultaneously, generally compensating one for another. Therefore, a new method that can achieve overall improvement need to be developed.

The atmospheric-pressure low-temperature plasma treatment has been shown to be a promising method for material performance enhancement, due to its advantages such as controllable, energy-efficient, and green^16–18^. It has been confirmed in some studies that multiple performances can be simultaneously enhanced by plasma-induced film deposition, which alters the physicochemical structure of the material. Chabane et al. deposited SiOF thin films using CF_4_/HMDSO/O_2_ plasma, which decreased the dielectric constant to 3.1 by enhancing Si-F bonds and reducing polarizability^19^. Zhu et al. treated defective Cu/epoxy resin interfaces with Ar/HMDSO plasma, depositing a Si-containing film that enhanced hydrophobicity and suppressed partial discharge (PD), resulting in a 38% increase in flashover voltage^20^. Chen et al. utilized He/CF_4_ plasma for EP surface fluorination, enhancing surface hydrophobicity by about 38.5%^21^. Unfortunately, the polymerized film often works as thermal barrier, reducing the thermal conductivity, which is unfavorable to the insulating paper stability^22,23^. The most effective strategy is to add thermally conductive fillers. Xiong et al. treated polyimide (PI) film with methyltrimethoxysilane (MTMS)/aluminum nitride (AlN), depositing a composite film that increased thermal conductivity to 1.68 W/(m·K)^7^. BN exhibits ideal thermal conductivity (λ > 600 W/(m·K)) for its layered structure, and the effectiveness of BN has been demonstrated in studies. Li et al. incorporated boron nitride (BN) into epoxy to enhance its thermal conductivity, which achieved a remarkable 204% increment compared to the pure epoxy resin^24^. Mun et al. mixed BN microspheres with polydimethylsiloxane (PDMS), resulting in a dramatic 2050% increase in thermal conductivity^25^. Therefore, plasma polymerization with BN addition has a broad prospect in improving electrical, thermal, and waterproof performances of paper.

In this paper, the insulating paper is treated by atmospheric-pressure low-temperature plasma, and the comprehensive enhancement is realized with mixture of Octamethylcyclotetrasiloxane (OMCTS) and BN as precursor. The thermal, electrical, waterproof and mechanical properties are evaluated with characteristic parameters, such as thermal conductivity, dielectric constant, flashover voltage, breakdown strength, WCA, water absorption, and tensile strength. The mechanism on the enhancement of oil-paper insulation performance is discussed combined with physicochemical characterization.

## Methods

### Plasma treatment

**Fig. 1:**
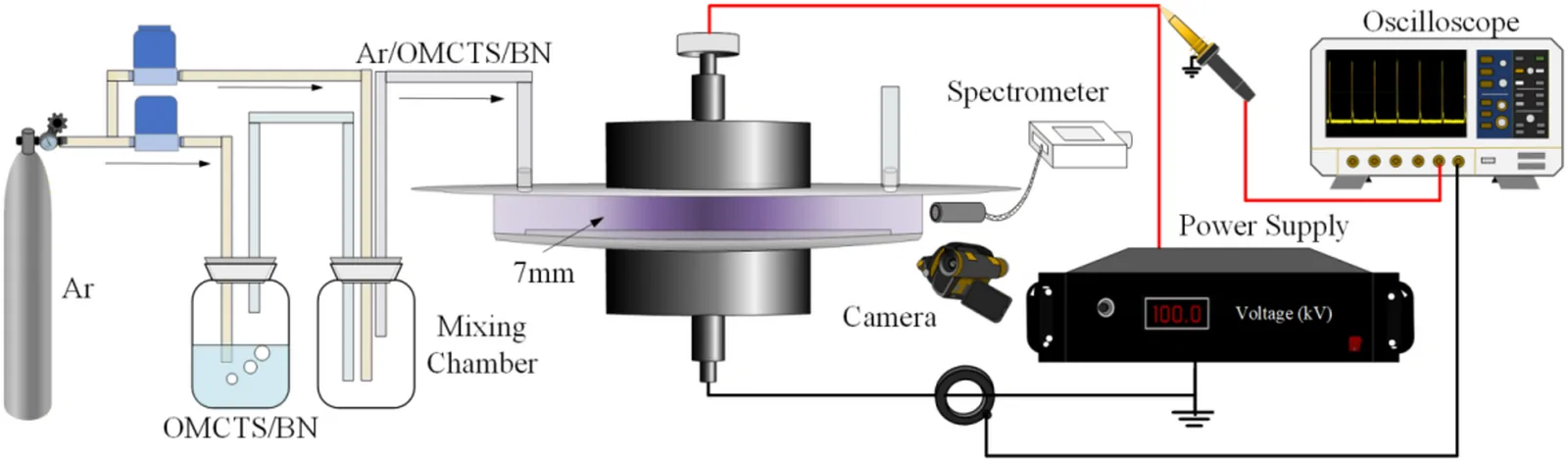
Experimental system.

**Fig. 2:**
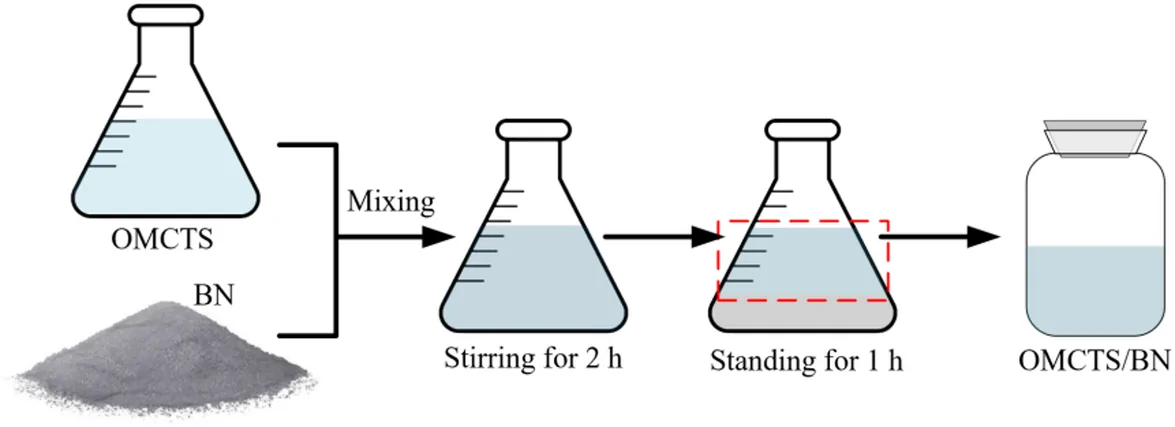
OMCTS/BN preparation method.

The experimental system of plasma treatment is presented in Fig. 1. A plate-plate electrode structure is employed to generate dielectric barrier discharge (DBD) with a quartz glass (1 mm thick) as the barrier. The neutral kraft paper, widely used in high voltage transformers, is utilized as the sample with 0.12 mm thickness (produced by Shuangfeng Company, China). It is composed of natural cellulose fibers, characterized by the density of 1.3 ± 0.1 g/cm^3^, thickness of 0.12 ± 0.007 mm, and ash content less than 1%. Ar is used as the working gas, while OMCTS (98%, Aladdin, China) and BN (ZY-BN-4, Zhongye New Material Technology Co. Ltd., China) are employed as precursors. The discharge is excited by a nanosecond pulsed power supply (ADW-20K10A, China). The power supply parameters are set as a pulse voltage of 12 kV, a repetition frequency of 5 kHz, a rising/falling edge of 100 ns, and a pulse width of 800 ns^20^. The voltage and current are measured with a probe (Tektronix P6015A, USA) and a current sensor (Monitor-2877, USA), which are recorded by an oscilloscope (Tektronix TDS 2014B, USA). The experiments are carried out at 20 °C with the humidity of 40%. As depicted in Fig. 2, mixture of OMCTS/ BN with the content of 10 mL/g is added to a tube and stirred for 2 h. After standing for 2 h, the mixture is carried out by Ar with bubbling method, maintaining a total gas flow rate of 1 L/min and a carrier gas of 175 mL/min.

**Fig. 3:**
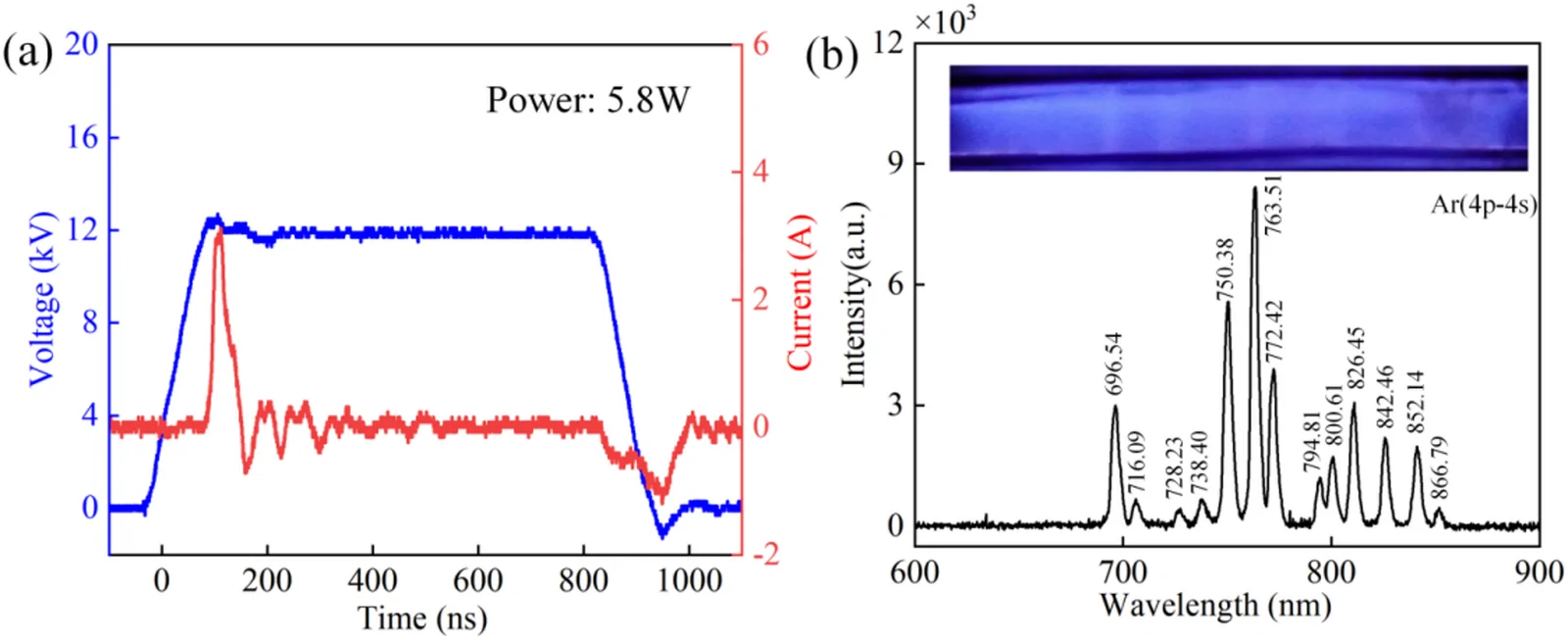
Characteristics of discharge plasma (**a**) voltage and current waveforms; (**b**) discharge images and OES.

**Fig. 4:**
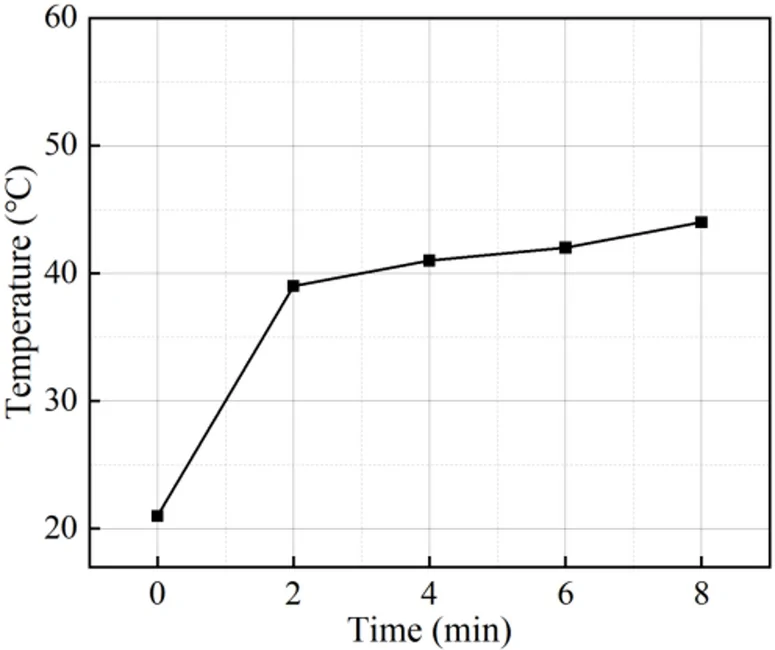
Variations of sample temperature with treatment duration.

The discharge plasma characteristics are illustrated in Fig. 3. Under these conditions, the discharge power of the DBD is 5.8 W, as depicted in Fig. 3(a). The uniform discharge is revealed throughout the entire gap from discharge image, and the OES spectrum is dominated by Ar (4p–4s). To determine whether the paper would undergo thermal degradation from the plasma’s heat during the treatment, the temperature of the sample corresponding to the bottom of the discharge region is measured by a thermal imaging camera (Fotric 325 Pro), and the results are shown in Fig. 4. Within treatment duration of 8 min, the internal temperature remains below 45 °C, far below the decomposition temperature of the insulating paper (approximately 300 °C).

### Physicochemical characterization

To analyze the variations of the physical characteristics for the paper after plasma treatment, the surface morphology is observed by field-emission scanning electron microscopy (SEM, JSM7800F, Japan). The Si ratios at different positions are detected by the energy-dispersive spectroscopy (EDS). The chemical groups are explored using fourier transform infrared spectroscopy (FTIR, PerkinElmer, USA). The chemical bond variation is determined by X-ray photoelectron spectroscopy (XPS, Kratos AXIS-ULTRA DLD, UK), and subsequently fitted using XPSPEAK41 software. The Si are fitted into multiple sub-peaks corresponding to different binding states. The fitting process is constrained by maintaining consistent Full Width at Half Maximum (FWHM) across all samples in the same batch.

### Cellulose insulating paper properties

**Fig. 5:**
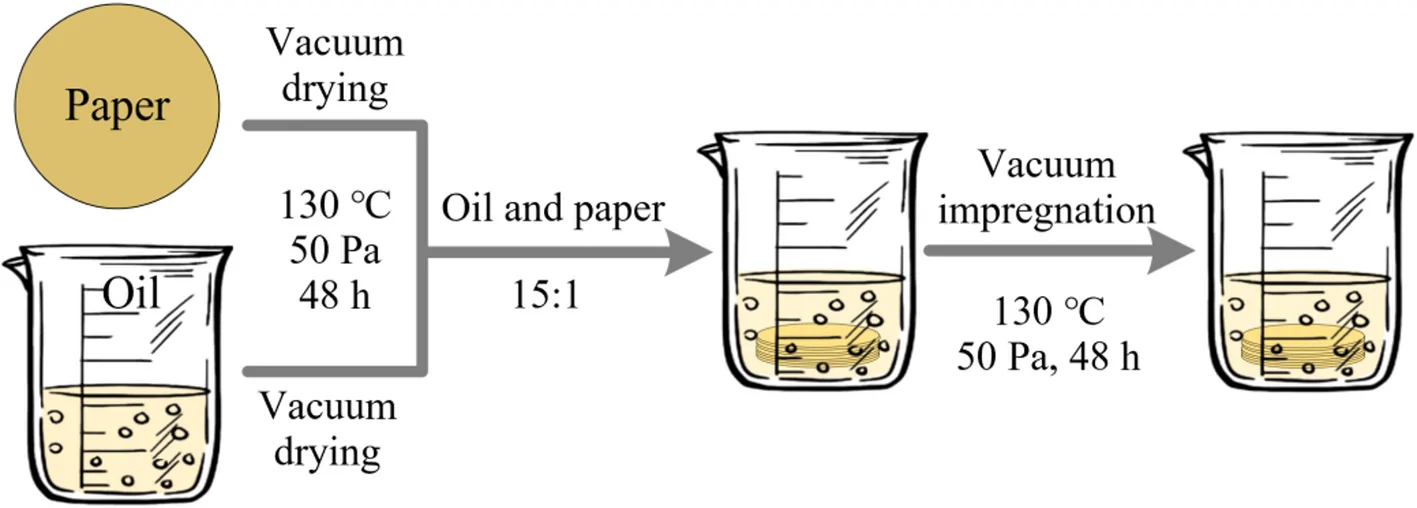
Preparation for oil-impregnated paper.

To evaluate the insulating paper performance enhancement, thermal, electrical, waterproof and mechanical properties are measured. The thermal conductivity is tested with an automatic instrument (DRL-V, China). The hot electrode temperature is set at 50 ℃ and the cold electrode temperature is 20 ℃. The pressure is applied to the paper at 200 N to minimize the contact thermal resistance. The thermal conductivity is calculated with Eq. (1)^26^

1 $$I=paC_p$$

where *p* is the density; *α* is the thermal diffusion coefficient; $C_p$ is the specific heat capacity. The paper capacitance is measured by a digital bridge (TH2811D, China), and the relative dielectric constant is calculated with Eq. (2)^27^

2 $$e_{r} = \frac{{4pkd}}{S}C$$

where *k* is the electrostatic force constant; *d* is the distance; *C* is the capacitance; *S* is the facing area. The voltage is applied following IEC 60,112. Initially, the voltage is increased at a rate of 0.5 kV/s until reaching 8 kV. Then the rate is reduced to 0.1 kV/s until the target is attained, which is maintained for 20 s. Each test condition is repeated fifteen times at different positions to ensure result reliability. The AC breakdown strength is evaluated with equal-diameter spherical electrodes. The voltage amplitude is ramped by rate of 0.5 kV/s^28^. For each treatment condition, fifteen samples are prepared and tested, with a 60 s interval between consecutive measurements to minimize experimental errors. The WCA is measured automatically (ZJ-CAZ2, China) with a 1.5 µL droplet. Water absorption is determined using the Cobb method, and the corresponding weight increment of the paper sample is recorded^29^. The water absorption is calculated with Eq. (3):

3 $$wt\% = \frac{{M_{p} - M_{c} }}{{M_{p} }} \times 100\%$$

where *M*_C_ and *M*_P_ are the mass before and after water absorption, respectively. The tensile strength is tested in accordance with the GB/T 12,914 standard, using an electronic tensile testing machine (CMT6103, China). PD measurements are carried out with a self-made platform^20^. PD signals and applied voltage signals are recorded with an oscilloscope (Tektronix, TDS-3054c, USA). Each acquisition captures 5 voltage cycles with peak detection sampling mode. To evaluate the oil-paper insulation performance, the papers are impregnated within mineral oil (Karamay 25#, 99.9%, China Petroleum and Chemical Corporation, China) following the process displayed in Fig. 5^30^.

## Results

### Physiochemical characteristics

**Fig. 6:**
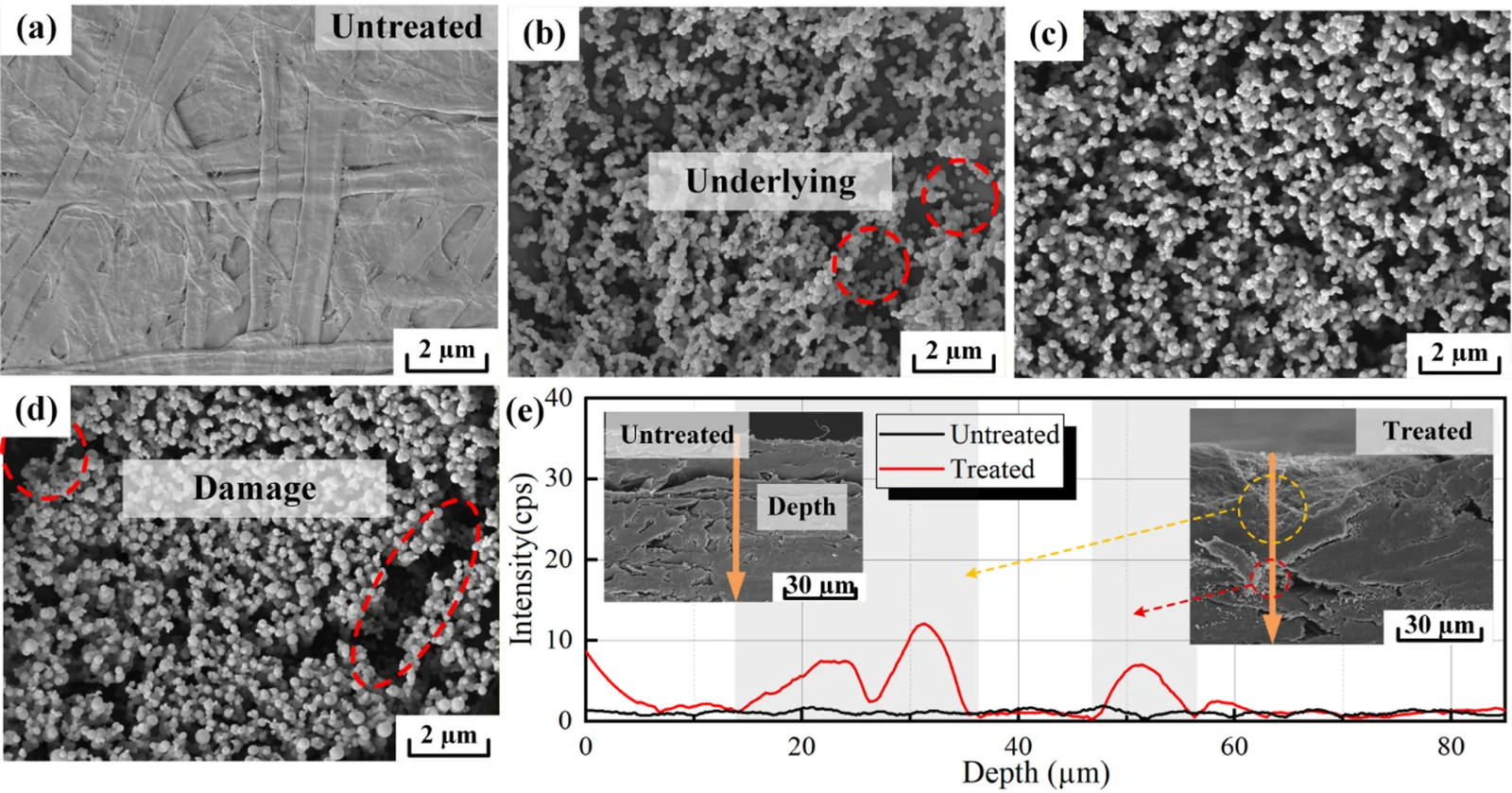
SEM results under different treatment durations (**a**) untreated; (**b**) 4 min; (**c**) 6 min; (**d**) 8 min; (**e**) Si intensity from EDS line scan of the cross section.

The variations in surface morphology before and after plasma treatment are presented in Figs. 6(a–d). The fibers on the untreated paper surface are stacked, forming a wrinkled structure, which ensures that the necessary mechanical robustness of the insulating paper is retained through their disordered intertwining. After plasma treatment, these fibers are covered by micro-scale clusters, as illustrated in Fig. 6(b–d). Specially, the original cellulose fibers are completely covered by a continuous film after 6 min treatment as depicted in Fig. 6(c). However, when the treatment duration is reduced to 4 min, the underlying paper is still partially visible due to insufficient polymerization. With over-treating for 8 min, the film integrity is damaged with distinct grooves. These effects on performance will be further analyzed in the following section. It should be noticed that the polymerization occurs not only on the surface but also within the bulk of the paper due to its porous structure. As indicated by Fig. 6(e), Trace amounts of silicon (less than 2 cps) are detected in untreated paper due to the manufacturing process. After plasma treatment, Si with an intensity exceeding 5 cps is appeared within a depth of 50 μm. It is demonstrated that Si-containing deposits are embedded in the paper, contributing to the improvement of the bulk properties of the insulating paper.

**Fig. 7:**
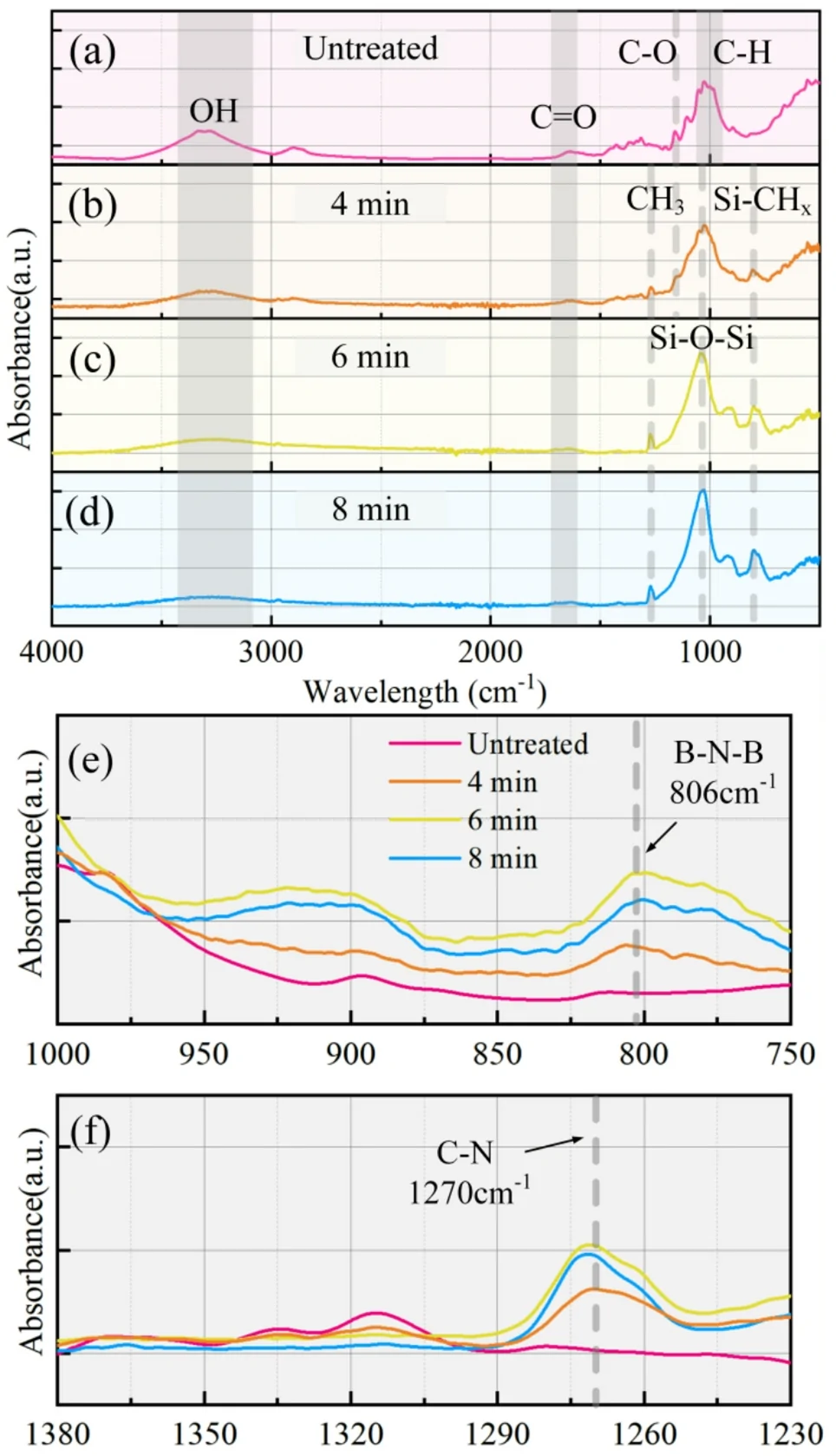
FTIR results under different treatment durations.

FTIR results are analyzed to reveal the chemical group variations. As displayed in Fig. 7(a–d), besides the inherent groups of the paper (such as OH, C=O), new radicals (such as Si–O–Si, Si–CH_x_) are observed, indicating plasma polymerization induced. The peak at 1051 cm^− 1^ is attributed to the asymmetric stretching vibration of Si–O–Si, while band at 851 cm^− 1^ is assigned to Si–CH_x_. An absorption band appears in the range of 1200–1500 cm^− 1^, corresponding to the symmetric bending of Si–CH_3_. The Si–O–Si and Si–CH_x_ are associated with the recombination of molecular fragments resulting from OMCTS dissociation^31–34^. Moreover, as depicted in Fig. 7(e) and (f), two characteristic peaks 806 cm^− 1^ and 1270 cm^− 1^ are detected, belonging to the B–N–B bending vibration and C–N stretching vibration, respectively, demonstrating that BN has been successfully participated in plasma polymerization. The chemical group proportions are changed with treatment durations, which will be further analyzed according to the XPS results.

**Fig. 8:**
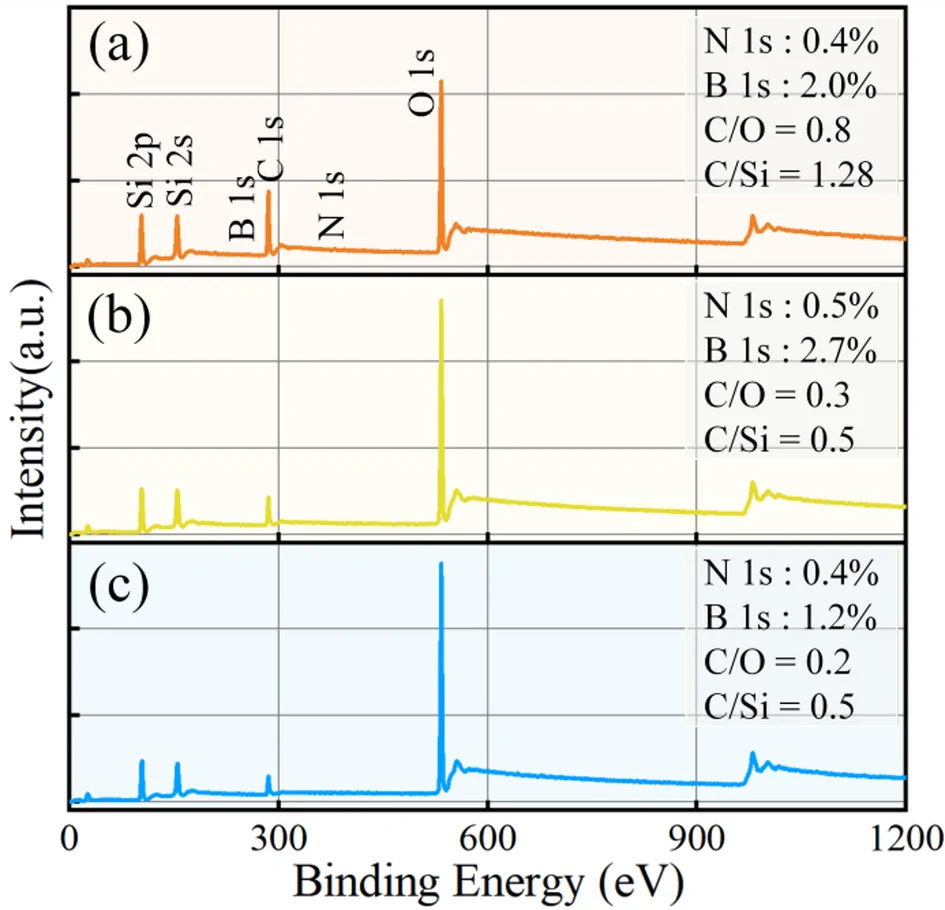
XPS full spectrum under different treatment durations (**a**) 4 min; (**b**) 6 min; (**c**) 8 min.

Figure 8 gives the XPS full-spectrum scanning of the insulating paper surface under different treatment durations. It is found that the presence of B and N elements is detected on the surface of paper after plasma treatment. As revealed by Fig. 8(b–d), ratios of both C/O and C/Si are gradually decreased with the treatment duration, demonstrating enhanced cross-linking of the organic silicon film. Each silicon atom is bonded to two methyl groups in the OMCTS molecule, yielding a theoretical C/Si ratio of 2. During plasma polymerization, energetic species preferentially cleave the relatively weak Si–C bonds, leading to the detachment of CH_3_ groups^35^. The resulting radical sites are subsequently recombined with reactive oxygen and silicon fragments to form a dense Si–O–Si network. Consequently, cross-linking of Si–O bone structure is enhanced as indicated by a decrease in the C/O and C/Si ratios. This structural evolution is further corroborated by the increasing dominance of Si_4/2_(Q) bonds.

**Table 1:** Elemental ratio in the XPS.

Condition	Elements ratio						
	Si	B	C	*N*	O	C/O	C/Si
4 min	25.1	2.0	32.0	0.4	40.5	0.8	1.3
6 min	29.0	2.7	15.0	0.5	52.8	0.3	0.5
8 min	27.1	1.2	13.3	0.4	58.1	0.2	0.5

**Fig. 9:**
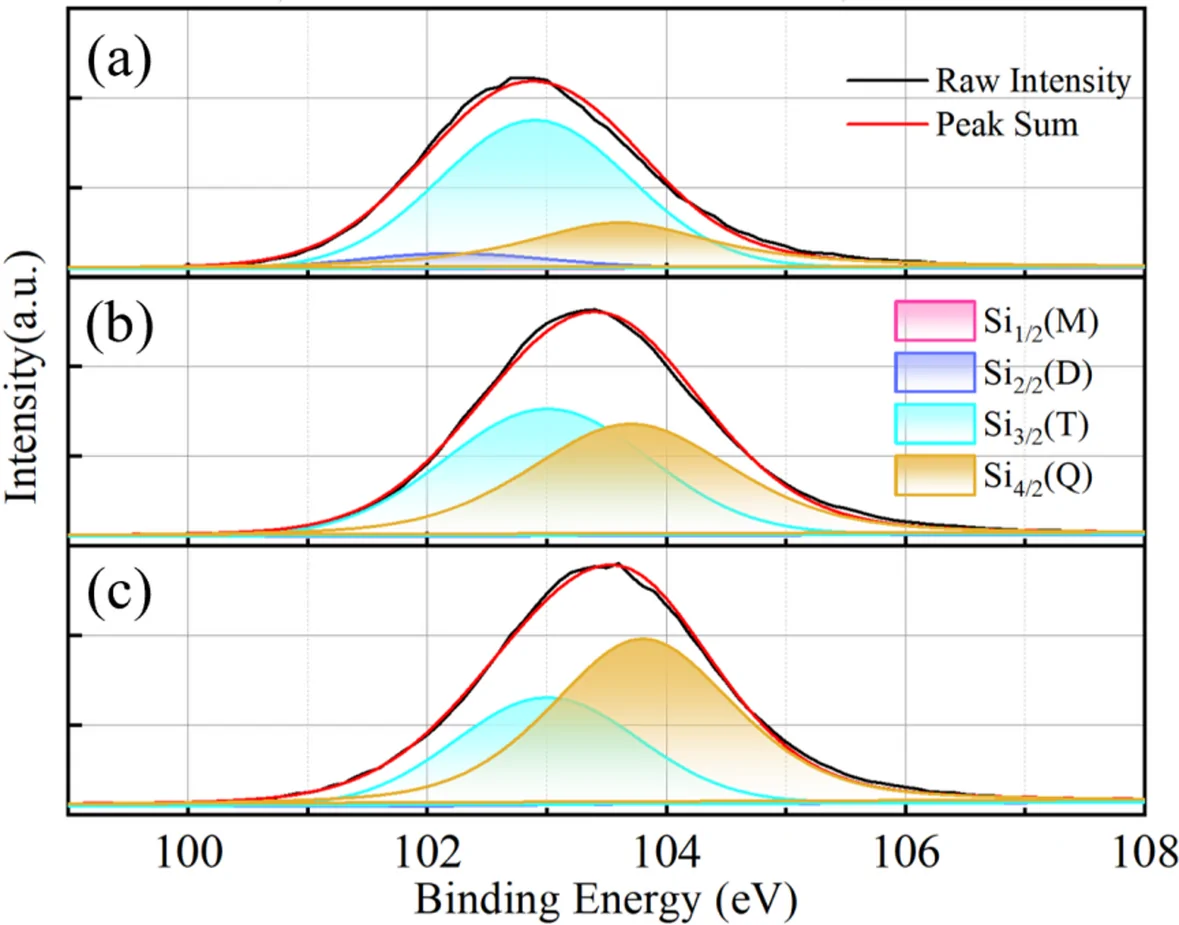
Si2p peak fitting results under different treatment durations (**a**) 4 min; (**b**) 6 min; (**c**) 8 min.

The Si2p peak and B1s fitting results under different treatment durations are given in Figs. 8 and 9, respectively. It is indicated that the chemical variation induced by plasma polymerization is significantly affected by the treatment duration. From Table 2,when the treatment duration is 4 min, the composition is dominated by Si_3/2_(T), which is 64.9%. When prolonged to 6 min, Si_4/2_(Q) becomes dominant, rising from 28.0% to 61.8%, while Si_3/2_(T) is decreased and Si_2/2_(D) is disappeared, indicating enhancement of cross-linking. When the treatment duration is increased to 8 min, the Si_4/2_(Q) is further raised to 71.9%, representing a further increase in the inorganic degree. It is demonstrated by the above that the extended treatment duration improves the degree of polymerization. It is for reason that the fiber is exposed to the plasma for a longer duration and polymerization reactions occur more frequently, leading to bond breaking of Si–CH_3_ and more Si–O–Si bonds formation, thus increasing cross-linking degree^36,37^.

**Table 2:** Variations of Si bond ratio under different treatment durations.

Condition	Si_1/2_(M)	Si_2/2_(D)	Si_3/2_(T)	Si_4/2_(Q)
Binding energy(eV)	100.9 ± 0.1	102 ± 0.1	102.9 ± 0.1	103.6 ± 0.1
4 min	0	7.1%	64.9%	28.0%
6 min	0	0	38.2%	61.8%
8 min	0	0	28.1%	71.9%

### Thermal performance

**Fig. 10:**
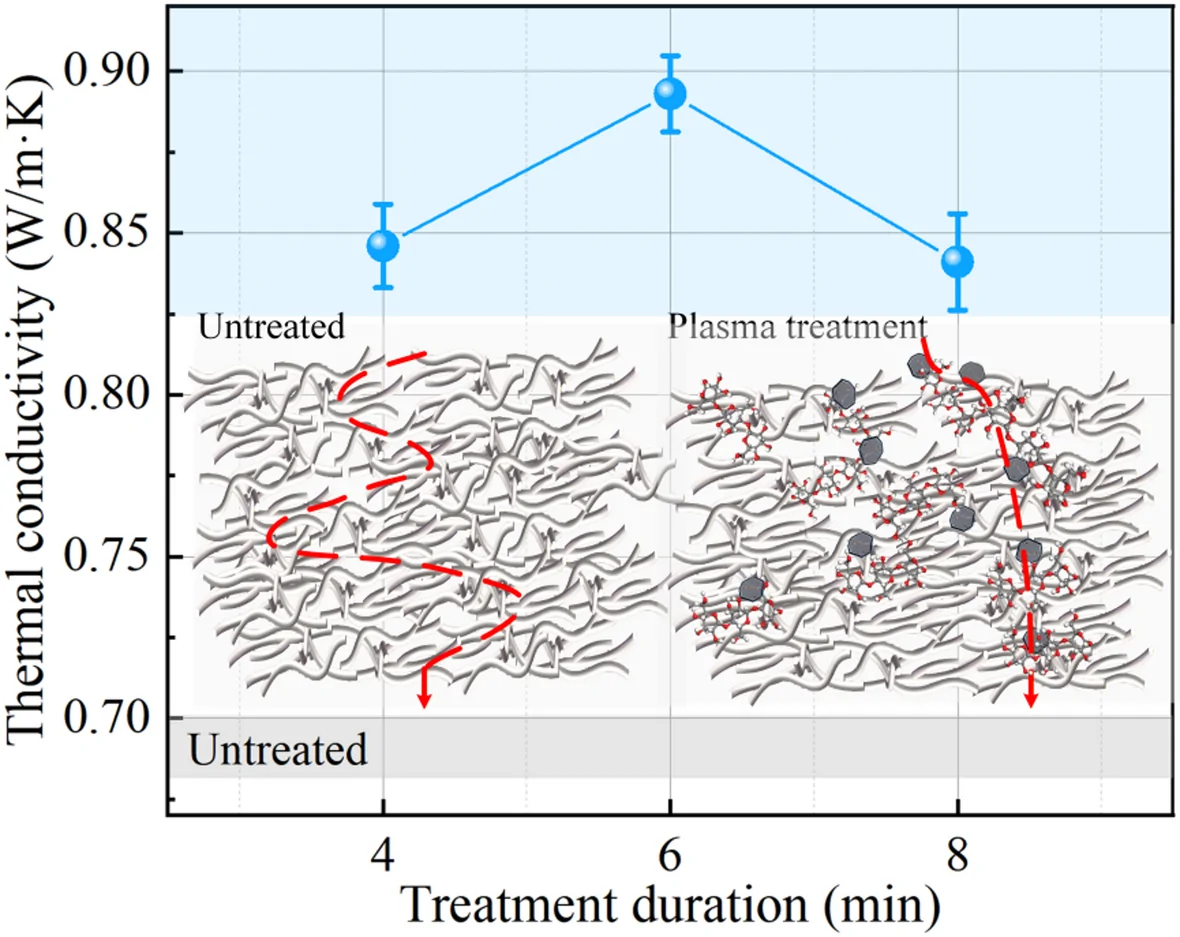
Thermal conductivity of the insulating paper under different treatment durations.

The thermal conductivity variations under different durations are depicted in Fig. 10. The thermal conductivity of the untreated is 0.69 W/(m·K), which is constricted by the tortuous and disordered fiber pathways for the phonon transport, leading to significant phonon scattering and diminished efficiency^22^. After plasma treatment, unique conductive pathways are generated facilitating the phonon transfer contributed by the addition of BN, thereby enhancing the thermal conductivity. The thermal conductivity is increased to 0.85 W/(m·K) under treatment duration of 4 min. When increasing to 6 min, it is increased to 0.89 W/(m·K), attributed to more BN introduction in the film as supported in Figs. 7 and 8. The formation of heat conduction path is promoted due to layered structure of BN, accelerating phonon transmission^24^. However, when further extending to 8 min, phonon migration may be restricted by damage on the film as indicated by Fig. 6(d), which will impact the heat transfer detrimentally, causing the thermal conductivity decreased to 0.84 W/(m·K).

### Electrical performance

#### Dielectric constant

**Fig. 11:**
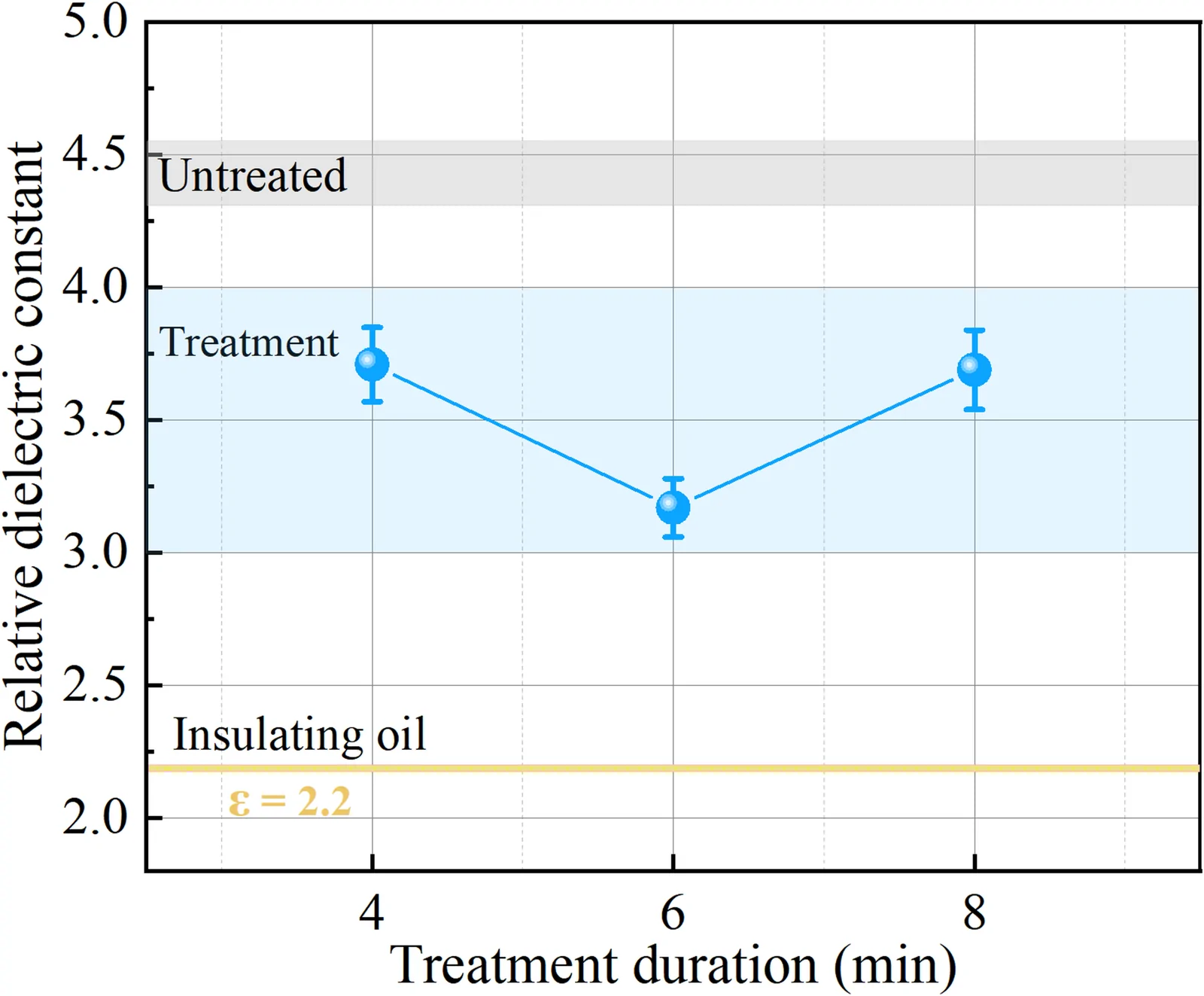
Relative dielectric constant variations of the insulating paper under different treatment durations.

Unreasonable electric field distribution within oil-paper insulation is predominantly caused by the mismatch in the dielectric constant of hybrid oil-paper insulation system. The relative dielectric constant of the insulating paper is 4.4, which is about two times higher than oil (ε ≈ 2.2), as depicted in Fig. 11. For untreated insulating paper, highly polarity -OH groups with a large dipole moment are present, which makes the molecules move readily under external electric field. After plasma treatment, the relative dielectric constant initially decreases and subsequently increases with treatment duration. When the treatment duration is 4 min, the relative dielectric constant is reduced to 3.7, attributed to low-polarity silicon groups bounded to cellulose molecules, such as Si-O-Si as shown in Fig. 7. When prolonged to 6 min, it is reduced to 3.2, 27.3% lower than untreated, since molecular orientation polarization is further suppressed by increasing Si_4/2_(Q) ratio, supported by Fig. 9(b). However, when extending to 8 min, the relative dielectric constant is increased to 3.7, which may be caused by the damage in Fig. 6(d), although the cross-linking degree is further increased^38^.

#### Surface insulation performance

**Fig. 12:**
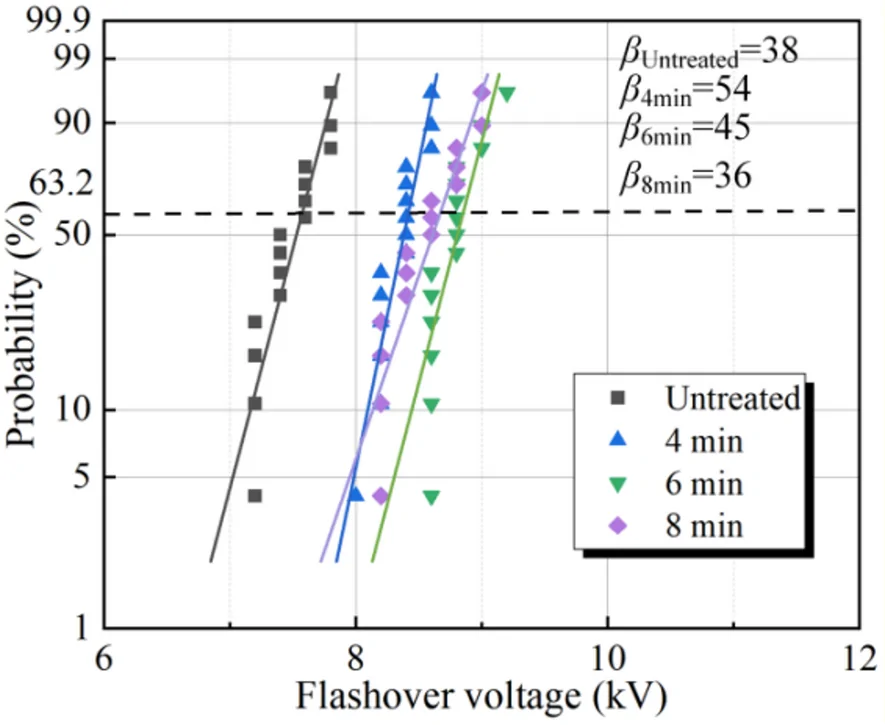
Flashover voltage of the insulating paper under different treatment durations.

The variation in flashover voltage under different treatment durations is presented in the Fig. 12. The flashover voltage of the untreated paper is 7.6 kV. After plasma treatment, the flashover voltage initially increases and then decreases with the treatment duration. When treatment duration is 4 min, the flashover voltage is increased to 8.4 kV attributed to the deposits that build a physical barrier as described in Fig. 6(b), to prolong the creepage distance and thus inhibit the charge mobility. When extended to 6 min, the flashover voltage is further enhanced to 8.9 kV owing to improved film continuity. However, when the treatment duration is 8 min, the physical damage, as depicted in Fig. 6(d), causes charge accumulation, thereby decreasing flashover voltage compared to that for the treatment of 6 min^39,40^. Furthermore, the *β* value is increased from 38 (untreated) to 54 (4 min) and 45 (6 min), indicating that the surface uniformity is improved after the treatment. However, the *β* value is decreased to 36 at 8 min, which aligns with the observation that excessive treatment causes surface damages in the SEM, introducing new local irregularities.

#### Bulk insulation performance

**Fig. 13:**
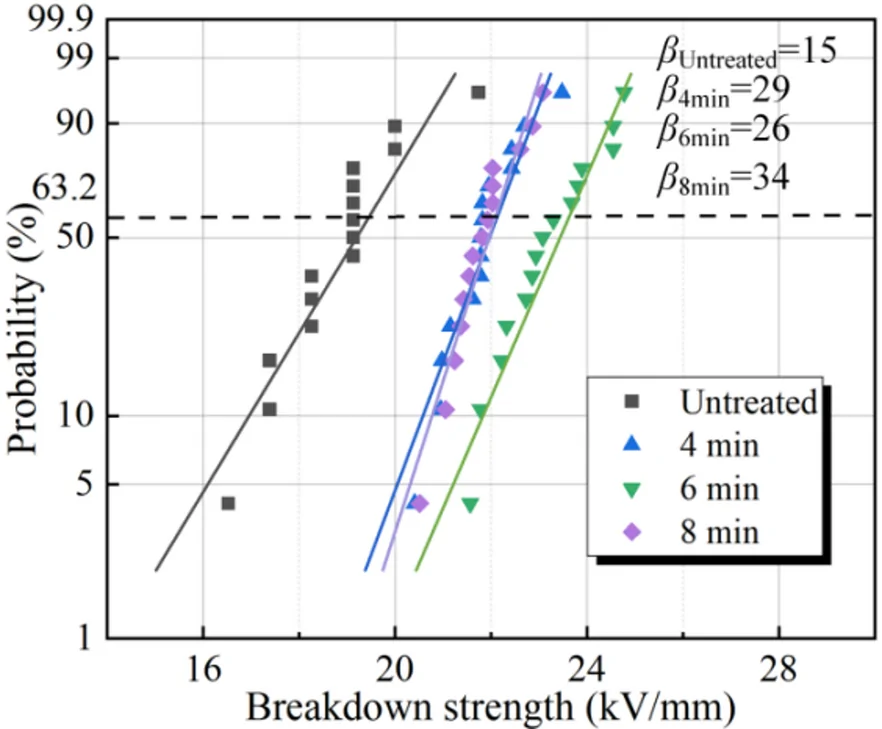
Breakdown strength under different treatment durations.

The influence of the treatment duration on the bulk insulation performance is evaluated with breakdown strength and PD characteristics. From Fig. 13, the breakdown strength is characterized by an initial enhancement followed by a subsequent decline with the treatment duration. The breakdown strength peaks at 23.8 kV/mm under treatment for 6 min, 21.9% higher than the untreated. This improvement can be explained in combination with the cross-sectional SEM and EDS. The silicon concentration decreases with depth is observed in Fig. 6(e), confirming that the polymerization of fragmented precursor molecules occurs within the paper. Therefore, the air gaps prone to insulation failure are filled, resulting in enhancement in breakdown strength. Furthermore, the *β* values of all treated samples are higher than that of the untreated paper. This improvement confirms that the penetration of plasma-activated fragments effectively fills the internal micro-pores and air gaps, homogenizing the bulk of the paper and reducing the randomness of breakdown.

**Fig. 14:**
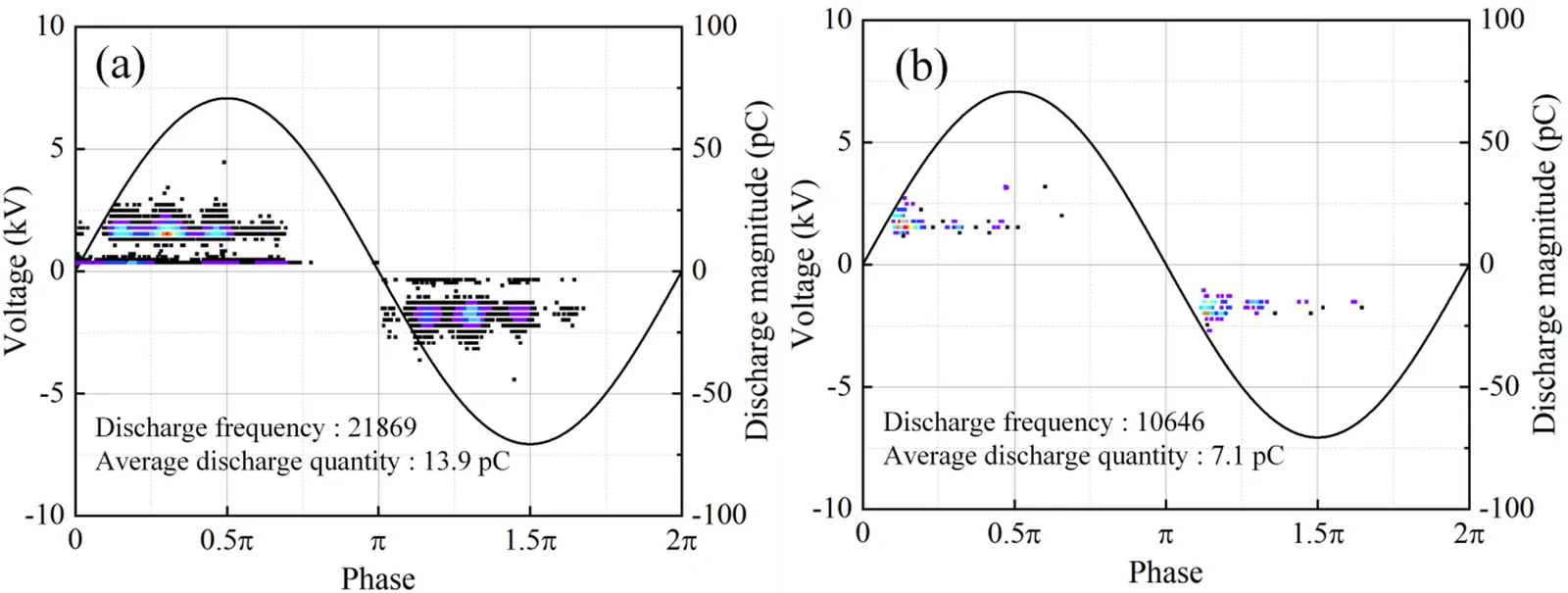
PD characteristics (**a**) untreated; (**b**) after treated for 6 min.

The PD characteristics under different treatment durations are shown in Fig. 14. PD for the untreated paper is measured with 21,869 discharges and an average discharge quantity of 13.9 pC. After the optimal treatment duration of 6 min, the number of discharges is reduced to 10,646, and the average discharge quantity is decreased to 7.1 pC, indicating that PD has been significantly inhibited. This is primarily attributed to the filling of internal voids and gaps by plasma polymerization, which restricts the ionization of air within the paper. Furthermore, as air pores are replaced by the Si–O–Si network, the local electric field distribution is homogenized, resulting in a lower probability of discharge within the insulating paper^20^.

### Waterproof performance

**Fig. 15:**
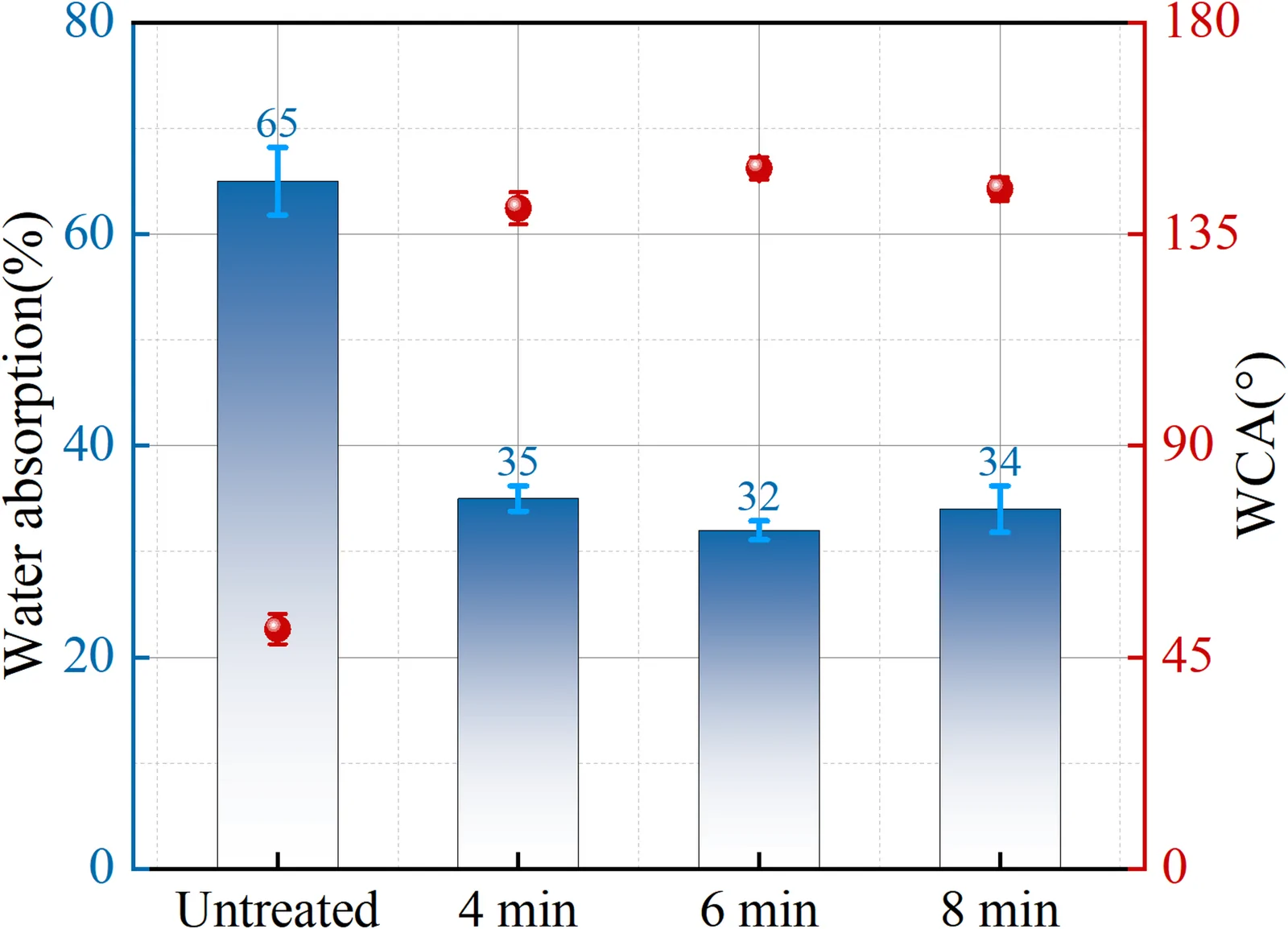
Waterproof performance under different treatment durations.

To evaluate the waterproof performance of paper, WCA and water absorption measurements are performed, as given in Fig. 15. The WCA of the untreated paper is 51° owing to the –OH bonds of the cellulose. After plasma treatment, the WCA is increased to over 140°, attributing to its low-polarity groups induced by plasma polymerization, such as CH_x_ and Si–O–Si. The water absorption of the untreated paper is measured at 65.1%, caused by the widespread distribution of micron-sized aperture channels and –OH bonds. The water absorption is reduced to less than 35.0% after plasma treatment. It can be explained that the internal micro-pits and grooves are filled by silicone-based polymers, which are characterized by low-polarity groups, suppressing the entry and transport of droplets from the surface into the bulk.

**Fig. 16:**
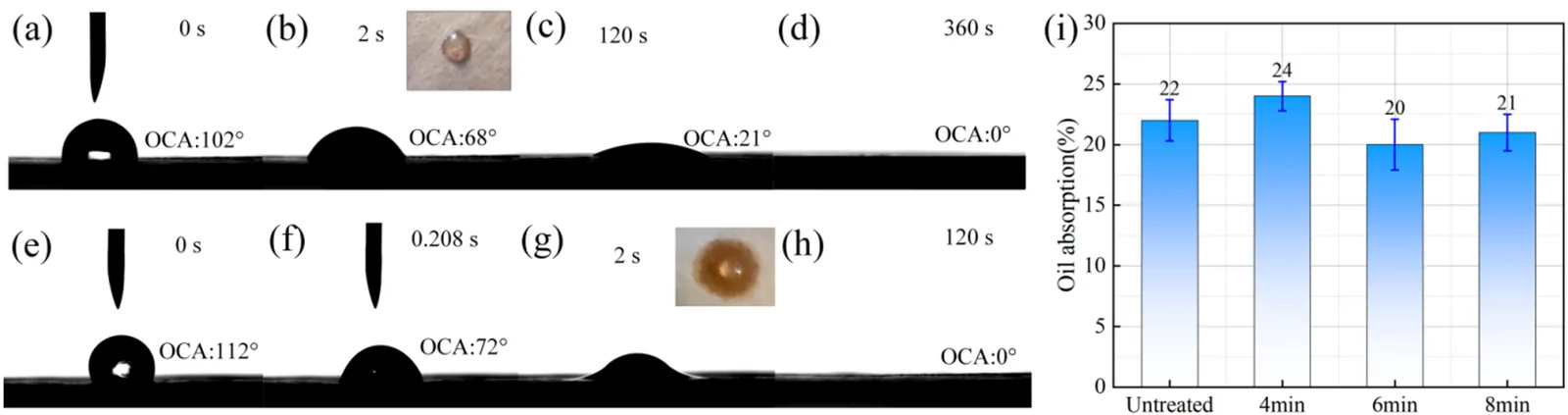
Variation of OCA with time (**a**)–(**d**) untreated; (**e**)–(**h**) plasma treatment for 6 min; (**i**) oil absorption.

The interaction between the oil and the paper is significantly altered, as the surface and internal voids are filled with the deposits during plasma treatment. To characterize the performance of oil impregnation before and after plasma treatment, the oil contact angle (OCA) is measured as presented in Fig. 16. Slow oil penetration (360 s) is observed for the untreated paper due to high interfacial energy caused by high-polarity -OH groups, while the OCA of paper treated for 6 min is decreased to 0° within 120 s, demonstrating superior lipophilic behavior. It is due to the fact that the oil penetration process is effectively accelerated by the surface rich in inorganic Si-O-Si groups, whereby minimizing the entrapment of air bubbles^41^. Consequently, a more uniform oil-paper structure is established. The enhancement is intrinsically ascribed to the contribution of the facilitated oil impregnation and pore filling. It is worth noting that the plasma-deposited film affects only the rate of oil absorption and has no impact on the ultimate oil saturation level, which remains at about 20%, as shown in Fig. 16(i).

### Mechanical performance

**Fig. 17:**
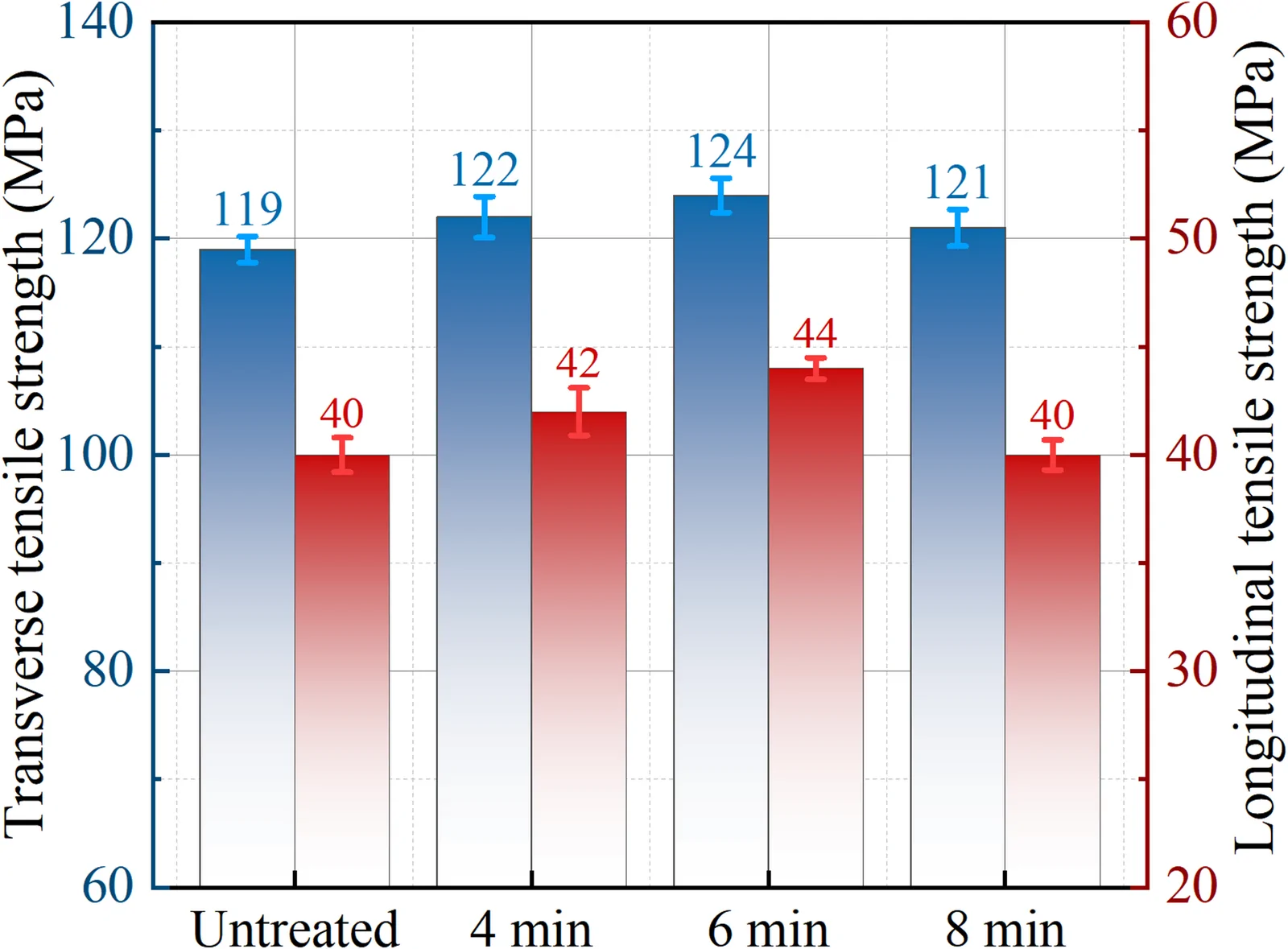
Mechanical strength under different treatment durations.

Variation of the tensile strength is depicted in Fig. 17. The transverse tensile strength of the paper remains essentially unchanged, while the longitudinal tensile strength is improved by 10.0% after optimal treatment duration of 6 min. As the plasma-activated fragments penetrate the surface into the paper, cross-linking polymerization occurs between the cellulose fibers. Therefore, more knots are formed in the treated insulating paper, so that the stress is decomposed into several component forces, resulting in the enhancement of the longitudinal tensile strength^42^.

### Oil-paper insulation

**Fig. 18:**
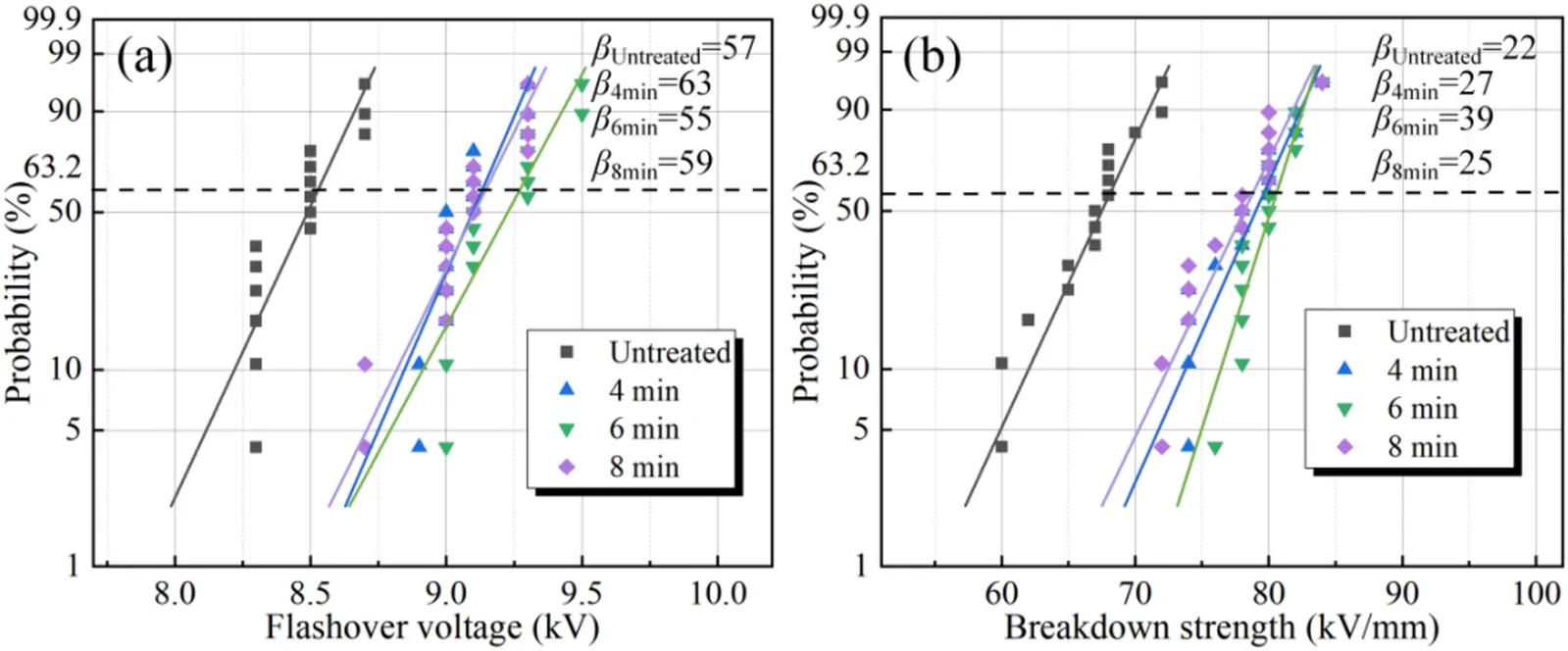
Oil-paper insulation performances under different treatment durations (**a**) flashover voltage; (**b**) breakdown strength.

The surface and bulk insulation performance of the oil-impregnated paper is enhanced by plasma treatment as illustrated in Fig. 18. For the untreated paper, a flashover voltage of 8.5 kV and a breakdown strength of 68.1 kV/mm are observed, increasing by 14.5% and 245.7%, respectively, compared with the non-impregnated paper. This improvement is owing to the occupation of internal pores and gaps by the insulating oil, through which excess air, impurities, and moisture are effectively expelled. Furthermore, the *β* values remain relatively stable across different treatment durations, indicating that the film combined with oil impregnation maintains surface homogeneity. In contrast, for breakdown strength, the *β* values of all treated samples are higher than that of the untreated oil-impregnated paper, peaking at the treatment of 6 min. It is confirmed that the synergistic effect of plasma-activated fragments penetrating the bulk and oil filling the micro-pores effectively homogenizes the internal structure, enhancing overall insulation reliability. Following optimal treatment duration of 6 min, an increment of 9.4% in flashover voltage and 17.5% in breakdown strength is achieved compared with the untreated oil-impregnated paper, facilitated by the deposition of active ingredients onto the surface and into the bulk, which will be further analyzed in discussion.

## Discussion

It is demonstrated that thermal, electrical, waterproof and mechanical performances of the cellulose insulating paper can be enhanced by plasma treatment with OMCTS/BN addition. The mechanism on the enhancement will be discussed in this section.

**Fig. 19:**
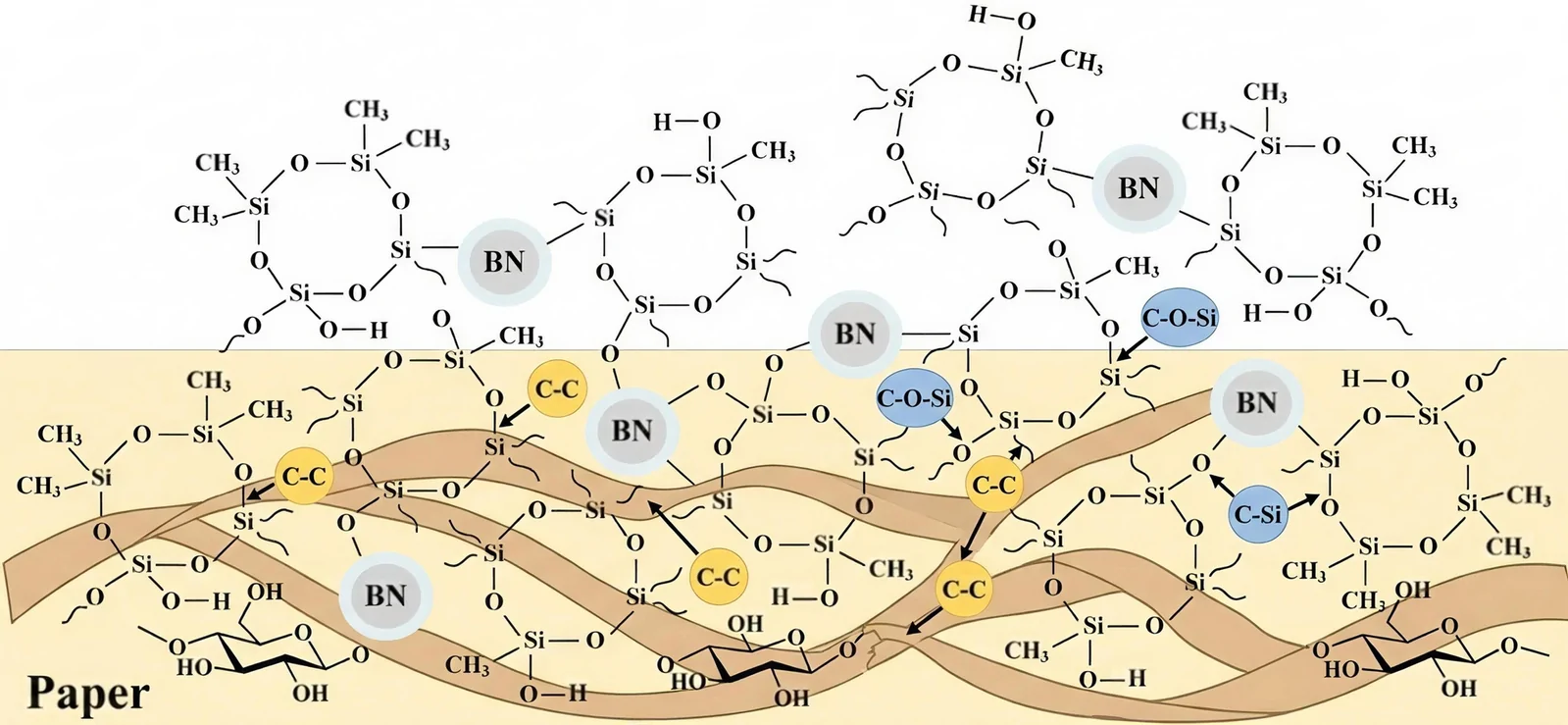
Illustration of plasma polymerization for the cellulose paper.

The plasma-activated OMCTS fragments undergo a process: they are deposited onto the paper surface to form a film, while simultaneously infiltrating the internal structure to integrate within the cellulose as shown in Fig. 6. During plasma treatment, the cellulose chains are attacked by high-energy electrons and ions, breaking chemical bonds (such as O–H and C–C) to generate abundant active sites on the fiber surface. These active cellulose radicals react with the OMCTS fragments, forming covalent bonds (e.g., C–O–Si or C–Si) at the interface. Simultaneously, as the Si–O–Si network undergoes highly cross-linked polymerization, it is wrapped around the cellulose fibers, acting as an inter-fiber binder. Moreover, with the addition of BN, bonding of -OH groups, which envelop the BN particles, enables the nanoparticles to embed in the Si–O–Si network tightly, producing a composite film structure, as depicted in Fig. 19. Therefore, the enhancement in thermal conductivity is facilitated by these BN inclusion, through which the heat dissipation is significantly accelerated. A significant reduction in the relative dielectric constant is achieved as low polarity silicon fragments (e.g. Si_3/2_(T) and Si_4/2_(Q)) penetrated into the insulating paper and bond with cellulose molecules, whereby reducing the molecular polarity. Both surface and bulk insulation performances are enhanced because physical defects and internal micro-voids are filled by the plasma polymerization. Through the synergistic effect of surface reinforcement and internal pore filling, the force is dispersed across the strengthened fiber knots, enhancing the mechanical strength. By incorporating low polarity groups, the hydrophobicity is improved. Furthermore, the interaction between H_2_O and the –OH groups within paper is hindered by the silicone deposits on the fiber surface, whereby the waterproof performance is enhanced.

**Fig. 20:**
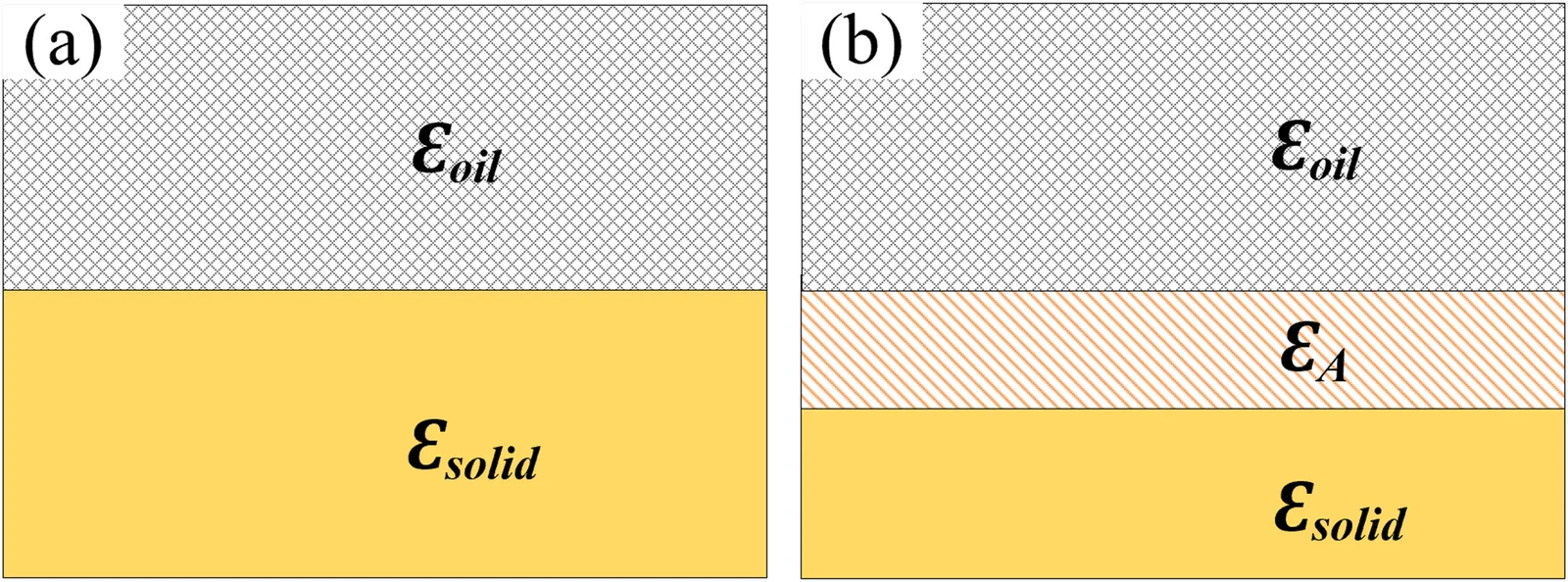
Equivalent schematic of dielectric model for oil-paper insulation system (**a**) untreated; (**b**) after plasma treatment.

The enhancement of oil-paper insulation is contributed by dielectric adaptation and oil impregnation. To explain the dielectric adaptation in oil-paper insulation systems after plasma treatment, the dielectric model is established as depicted in Fig. 20. The oil-impregnated paper is a kind of hybrid material composed by solid fibers and liquid oil, as shown in Fig. 20(a). After plasma treatment, polymerization reactions are induced on cellulose molecules to form a cross-linking organosilicon network with high proportion of low-polarity Si_4/2_(Q), which suppresses molecular orientation polarization, making dielectric constant (*ε*_*A*_) lower than pure paper (*ε*_*solid*_). Therefore, dielectric adaptation between the modified cellulose paper and the surrounding oil is achieved, weakening electric field distortion.

## Conclusion

In summary, plasma with OMCTS/BN as the precursor is demonstrated as an effective approach to polymerize on the surface and within the bulk of the cellulose insulating paper, enhancing its thermal, electrical, waterproof, and mechanical performances. The main conclusions are drawn as follows:

- The thermal conductivity of the insulating paper is increased from 0.69 W/(m·K) to 0.89 W/(m·K) under the optimal treatment duration of 6 min, which is contributed by the promoted heat conduction path with BN.
-  A high proportion of Si_4/2_(Q) is introduced to suppress molecular polarization and reduce the relative dielectric constant. After oil impregnation, enhancement in insulation performance is exhibited for the treated insulating paper due to dielectric adaptation and lipophilic behavior.
- The WCA is increased from 51° to approximately 150°, and water absorption is reduced to 32.6%, effectively inhibiting moisture ingress.
-  Penetration of plasma-activated fragments into the paper bulk strengthens fiber junctions, resulting in a 10.0% increase in longitudinal tensile strength.

Overall, this study provides a pathway for developing high-performance cellulose insulating paper for reliable oil-paper insulation systems.

## Data Availability

The datasets used and analysed during the current study are available from the corresponding author on reasonable request.
